# Cyclohexane Vibronic States: A Combined VUV Spectroscopy and Theoretical Study

**DOI:** 10.3390/molecules30071493

**Published:** 2025-03-27

**Authors:** Edvaldo Bandeira, Alessandra S. Barbosa, Nykola C. Jones, Søren V. Hoffmann, Márcio H. F. Bettega, Paulo Limão-Vieira

**Affiliations:** 1 Departamento de Física, Universidade Federal do Paraná, Caixa Postal 19044, Curitiba 81531-980, PR, Brazil; bandeira@fisica.ufpr.br (E.B.); alessandra@fisica.ufpr.br (A.S.B.); 2 ISA, Department of Physics and Astronomy, Aarhus University, Ny Munkegade 120, DK-8000 Aarhus C, Denmark; nykj@phys.au.dk (N.C.J.); vronning@phys.au.dk (S.V.H.); 3 Atomic and Molecular Collisions Laboratory, Centre of Physics and Technological Research (CEFITEC), Department of Physics, NOVA School of Science and Technology, Universidade NOVA de Lisboa, 2829-516 Caparica, Portugal

**Keywords:** cyclohexane, cross-sections, theoretical calculations, spectroscopy

## Abstract

In this work, we provide results from a joint experimental and theoretical study of the vibronic features of cyclohexane (C_6_H_12_) in the photon energy range of 6.8–10.8 eV (182–115 nm). The high-resolution vacuum ultraviolet (VUV) photoabsorption measurements, together with quantum chemical calculations at the time-dependent density functional theory (TDDFT) level, have helped to assign the major electronic excitations to mixed valence–Rydberg and Rydberg transitions. The C_6_H_12_ photoabsorption spectrum shows fine structure which has been assigned to CH_2_ scissoring, $v_{3}^{\prime }\left(a_{1g}\right)$, CH_2_ rocking, $v_{4}^{\prime }\left(a_{1g}\right)$, C–C stretching, $v_{5}^{\prime }\left(a_{1g}\right)$, and CCC bending/CC torsion, $v_{24}^{\prime }\left(e_{g}\right),$ modes. Molecular structure calculations at the DFT level for the neutral and cationic electronic ground-states have shown the relevant structural changes that are operative in the higher-lying electronic states. Photolysis lifetimes in the Earth’s atmosphere are shown to be irrelevant, while the main atmospheric sink mechanism is the reaction with the ^•^OH radical. Potential energy curves have been obtained at the TDDFT level of theory, showing the relevance of interchange character mainly involving the CH_2_ scissoring, $v_{3}^{\prime }\left(a_{1g}\right),$ and CH_2_ rocking, $v_{4}^{\prime }\left(a_{1g}\right),$ modes, while Jahn–Teller distortion yields weak vibronic coupling involving the non-totally symmetric CCC bending/CC torsion, $v_{24}^{\prime }\left(e_{g}\right),$ mode.

## 1. Introduction

Cyclohexane, C_6_H_12_, is a chemical compound produced from the hydrogenation of C_6_H_6_, and it has been used as a fuel constituent in the combustion process, hence playing a role in vehicle emissions [1,2,3,4]. C_6_H_12_ is a non-polar solvent widely used as an intermediate in the polymer industry, giving rise to significant emissions to the environment, as well as pollution reported from spills of petroleum products and the use of solvents [4], just to mention a few. It also contributes to the lower atmosphere local chemistry, mainly through reactions with ^•^OH radicals [2,5,6], and can undergo oxidation processes yielding alkoxy radicals [5,7,8,9,10], among others. The main atmospheric implications of C_6_H_12_ are related to its ability to act as an ^•^OH radical scavenger while reacting with other chemicals, e.g., Cl and O_3_ [5], thus relevant for consideration in modeling the atmospheric concentrations of volatile organic compounds (VOCs) [1]. Cyclohexane has been widely investigated by the international community regarding the identification of effective processes to remove it as a toxic pollutant. Recently, Dahiru et al. [4] reported the use of non-thermal plasmas for the reduction of cyclohexane at room temperature and under atmospheric pressure.

Over the last two decades, we have been interested in investigating the role of some VOCs when released in the Earth’s atmosphere [11,12,13,14,15,16]. To accomplish this purpose, we have produced comprehensive descriptions of the electronic state spectroscopy of such chemical compounds by recording high resolution photoabsorption spectra in the vacuum ultraviolet energy region, viz. 115–330 nm, together with the help of quantum chemical calculations. From the absolute values of the cross-sections above 180 nm, photolysis rates have been estimated from sea level (0 km) up to the limit of the stratopause (50 km altitude) (see [17] and the references therein). This joint experimental and theoretical effort is now being employed for C_6_H_12_ in the wavelength region investigated in this work, i.e., from 115 nm (10.8 eV) up to 182 nm (6.8 eV).

The assignment of different absorption features requires detailed and complementary information from other spectroscopic techniques, whenever available. We note previous work on electron impact ionization [18,19,20], photoelectron spectroscopy [21,22], photoionization [21,22], and Penning ionization [23]. Other examples include the VUV photoabsorption with no complete assignments in the energy region of 5.5–31.0 eV [21,24,25,26,27] and infrared and Raman spectroscopies [28,29,30]. Finally, the modeling of atmospheric concentrations and rates of reaction with ^•^OH radicals have been reported [1,2,5].

Section 2 deals with the structure and properties of cyclohexane, while Section 3 comprises the results and discussion, with a detailed description of the major electronic transitions and the fine structure assignments. Section 4 is devoted to a brief description of the experimental and theoretical methods. We close in Section 5 with the major conclusions from the present joint investigation of the electronic state spectroscopy of C_6_H_12_.

## 2. Structure and Properties of Cyclohexane

The total energy values with zero-point correction computed at the DFT/CAMB3LYP/aug-cc-pVTZ level of theory for cyclohexane conformers in the neutral ground-state and in the cationic ground-state show the *chair* and *boat* conformers, respectively, to be the most stable at room temperature (Table S1 and Figures S1 and S2).

**Figure 1:**
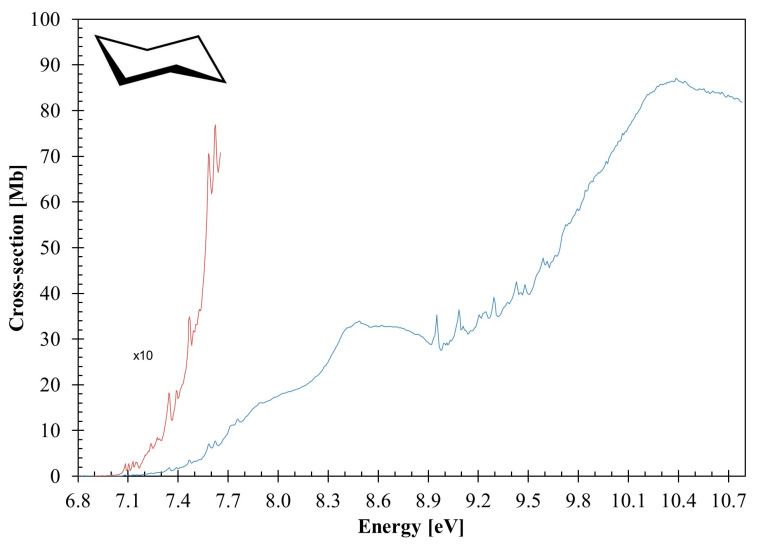
The photoabsorption spectrum of cyclohexane in the 6.8–10.8 eV photon energy range. See text for details.

**Figure 2:**
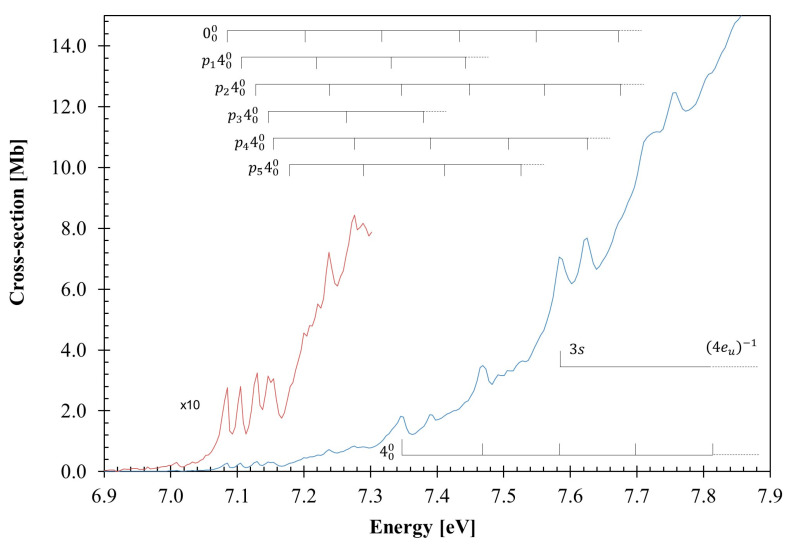
Detail of the photoabsorption spectrum of cyclohexane in the 6.9–7.9 eV photon energy range. See text for details.

**Figure 3:**
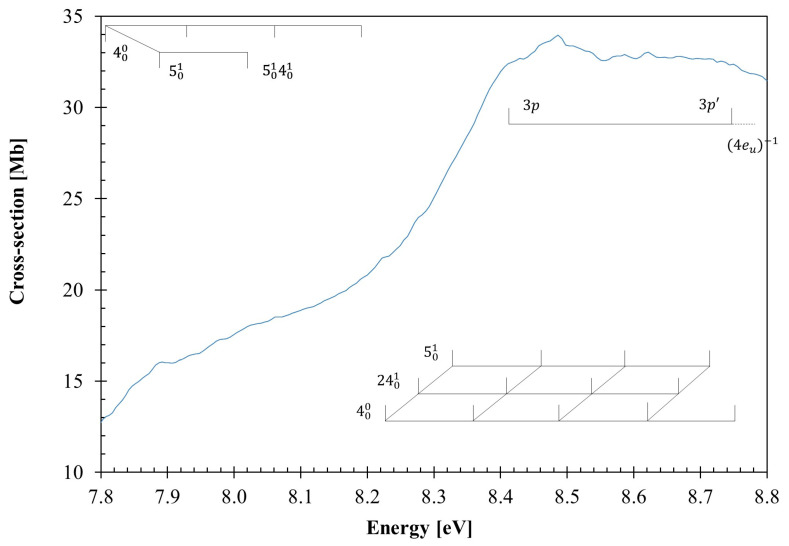
Photoabsorption spectrum of cyclohexane in the 7.8–8.8 eV photon energy range. See text for details.

**Figure 4:**
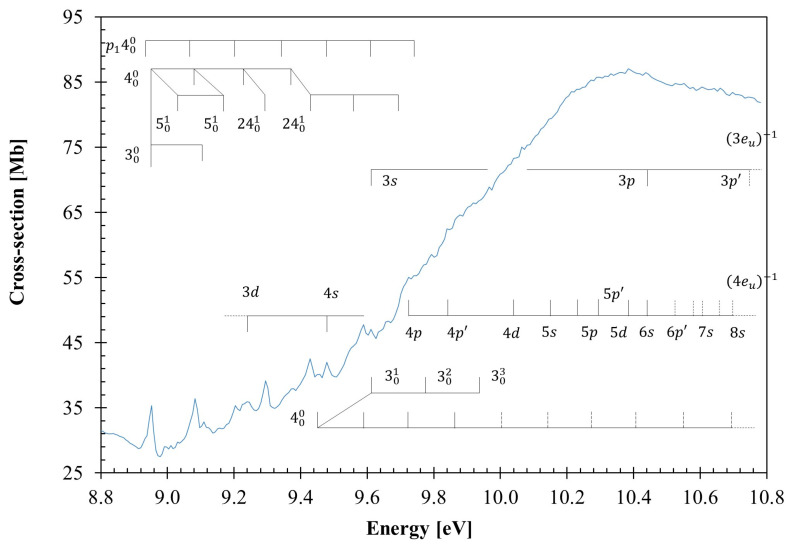
Photoabsorption spectrum of cyclohexane in the 8.8–10.8 eV photon energy range. See text for details. The vertical dashed lines show the tentative assignments of less-resolved features.

The calculated outermost electronic configuration of the X˜A1g1 ground-state is (3e_u_)^4^ (1a_1u_)^2^ (4e_u_)^4^ (4a_1g_)^2^ (4e_g_)^4^. The dominant transitions in the absorption spectrum (Figure 1, Figure 2, Figure 3 and Figure 4, Table 1 and Table S2) have been assigned to electronic (and vibrational) excitations (Table 2, Table 3 and Table 4) involving the (HOMO), 4e_g_, $\sigma_{CC/CH}$, the (HOMO-1), 4e_g_, $\sigma_{CC/CH}$, the (HOMO-2), 4a_1g_, $\sigma_{CH}$, and the (HOMO-6, HOMO-7), 3e_u_, $\sigma_{CH}$, to mixed valence–Rydberg and Rydberg character orbitals (Table 1). The fine structures in the photoabsorption energy region 6.8–10.8 eV are assigned to vibrational modes from Raman and infrared spectroscopies [28,29,30], together with the calculated harmonic frequencies for the neutral electronic ground-state (Table S3) and the cation electronic ground-state (Table S4). The main modes have been assigned in the neutral electronic ground-state to 0.182 eV (1465 cm^−1^) for CH_2_ scissoring, $v_{3}^{\prime \prime }\left(a_{1g}\right)$, 0.144 eV (1157 cm^−1^) for CH_2_ rocking, $v_{4}^{\prime \prime }\left(a_{1g}\right)$, 0.099 eV (802 cm^−1^) for C–C stretching, $v_{5}^{\prime \prime }\left(a_{1g}\right),$ and 0.053 eV (426 cm^−1^) for CCC bending/CC torsion, $v_{24}^{\prime \prime }\left(e_{g}\right)$ [31].

Table 5 lists the tentative assignments of the different Rydberg series based on their quantum defects. These have been obtained based on the lowest ionization vertical energies (IE_v_) available from the photoelectron spectroscopy experiments of Kovac and Klasinc [32] to be (IE_3_)_v_ = 10.98 (4e_u_)^−1^ and (IE_5_)_v_ = 13.03 eV (3e_u_)^−1^. Rydberg series converging to (4e_g_)^−1^, (4a_1g_)^−1^ and (4a_1u_)^−1^ are dipole-forbidden within the molecule’s *D*_3d_ group symmetry and are not assigned.

## 3. Discussion

The VUV photoabsorption spectrum of C_6_H_12_ is shown in Figure 1 in the energy range 6.8 to 10.8 eV, while the fine structure and the Rydberg assignments are shown in the enlarged sections in Figure 2, Figure 3 and Figure 4 (see also Table 2, Table 3, Table 4 and Table 5). The major absorption bands have been assigned to electronic excitations from the ground-state to mixed valence–Rydberg and Rydberg molecular orbitals with the help of the TDDFT/CAMB3LYP/aug-cc-pVTZ calculations in Table 1. The results of the calculations also include the vertical excitation energies and oscillator strengths and are compared with the experimental data.

The Rydberg character is related to each series, converging to the different ionic electronic states ${\left(4e_{u}\right)}^{-1}$ B˜Eu2 and ${\left(3e_{u}\right)}^{-1}$ D˜Eu2 (see Section 3.4). At a glance, the absorption bands are rather broad, and the spectrum baseline is continuously shifted above the baseline as the photon energy increases. The dominant electronic excitations in Table 1 are Rydberg, and only mixed valence–Rydberg character is noted above 9.9 eV. However, a close inspection of the complete set of electronic transitions (Table S2) and the representation of cyclohexane molecular orbitals (Figure S3) show that while the character of the unoccupied molecular orbitals is mostly Rydberg, there are minor contributions from $\sigma_{CC}^{*}$ and $\sigma_{CH}^{*}$ antibonding MOs. Thus, the latter may render an underlying dissociative character of the electronic transitions to the photoabsorption cross-section.

The fine structure noted throughout the photoabsorption spectrum in the energy range of 6.8–10.8 eV (Figure 1, Figure 2, Figure 3 and Figure 4) has been assigned to the contribution of the CH_2_ scissoring, $v_{3}^{\prime }\left(a_{1g}\right)$, the CH_2_ rocking, $v_{4}^{\prime }\left(a_{1g}\right)$, the C–C stretching, $v_{5}^{\prime }\left(a_{1g}\right),$ and the CCC bending/CC torsion, $v_{24}^{\prime }\left(e_{g}\right),$ modes. A detailed assignment of this structure, associated with the electronic transitions from Table 1, are listed in Table 2.

The results from the calculations in Table 1 show a good level of agreement with the experimental data for all electronic transitions to within 3%; however, the major difference is noted for the lowest-lying electronic transition, where this value increases to ~7%. The calculations have been performed in the *C*_2h_ symmetry group and correlating with the *D*_3d_ point group as $A_{u}⊕B_{u}\to \;E_{u}$ and $B_{u}\to \;A_{2u}$ for the dominant excitations. The next section contains a detailed description of the electronic (and vibrational) excitations, with the major transitions and their origins tentatively assigned, absolute cross-section values compared with others in the literature, and discussion about cyclohexane photolysis.

### 3.1. The 7.0–7.9 eV Photon Energy Range

The lowest-lying valence excitation is assigned to an electron promotion from the degenerate HOMO/HOMO-1, 4e_g_, $\sigma_{CC/CH}$ orbital to the LUMO+1 Rydberg molecular orbital (see Table 1 and Table S2, and Section 3.4),  3p 4a2u←σCC/CH4eg, 1Eu1←X˜A1g1, with a cross-section value of 3.49 Mb at 7.46(9) eV (Figure 2). The calculated vertical excitation energy of 8.026 eV and an oscillator strength *f*_L_ ≈ 0.00017 are ~7% higher than the experimental value. The $0_{0}^{0}$ origin is tentatively assigned at 7.085 eV, while the VUV spectrum of Raymonda [26] shows a feature at 7.005 eV (56,500 cm^−1^), showing up to five progressions of the CH_2_ rocking, $v_{4}^{\prime }\left(a_{1g}\right),$ mode, as listed in Table 2. Moreover, some of the features can be assigned to combinations of the CH_2_ scissoring, $v_{3}^{\prime }\left(a_{1g}\right)$ mode, while the C–C stretching, $v_{5}^{\prime }\left(a_{1g}\right)$ mode can also contribute to the spectrum. To avoid the congestion of Figure 2, $v_{3}^{\prime }\left(a_{1g}\right)$ and $v_{5}^{\prime }\left(a_{1g}\right)$ were not included, but the different energy values can be obtained from Table 2. The average spacings of $v_{3}^{\prime }\left(a_{1g}\right)$, $v_{4}^{\prime }\left(a_{1g}\right),$ and $v_{5}^{\prime }\left(a_{1g}\right)$ are 0.150 eV (1210 cm^−1^), 0.115 eV (936 cm^−1^), and 0.098 eV (790 cm^−1^), respectively. The feature at 7.583 eV is assigned to the first member of a Rydberg series converging to the ${\left(4e_{u}\right)}^{-1}$ B˜Eu2 ionic electronic state, which will be discussed in Section 3.4.

The second absorption band tentatively centered at 8.02(0) eV, with a maximum cross-section of 17.99 Mb, is assigned to the Rydberg excitation (Section 3.4) 3p/3p′ 5eu←σCC/CH4eg, 1A2u1←X˜A1g1, with an oscillator strength of ≈0.00201 (Table 1). The $0_{0}^{0}$ origin band is assigned at 7.345 eV (Table 2 and Figure 2) from its broadness related to a vibrational excitation, as well as from the shape of its left-hand side, showing a different slope from that of the former electronic state’s features. This band shows broad features, which are due to CH_2_ rocking, $v_{4}^{\prime }\left(a_{1g}\right)$ mode, and combinations with CH_2_ scissoring, $v_{3}^{\prime }\left(a_{1g}\right)$ mode, with average spacings of 0.153 eV (1234 cm^−1^) and 0.120 eV (968 cm^−1^), respectively.

### 3.2. The 7.8–8.8 eV Photon Energy Range

The photoabsorption spectrum in this energy range includes two electronic transitions centered at 8.07(7) and 8.486 eV (Figure 3), which are assigned to Rydberg transitions, with cross-section values of 18.57 and 33.96 Mb (Table 1 and Figure 3). The $0_{0}^{0}$ origin bands are assigned at 7.80(8) and 8.22(7) eV and are accompanied by excitation of CH_2_ rocking, $v_{4}^{\prime }\left(a_{1g}\right),$ and C–C stretching, $v_{5}^{\prime }\left(a_{1g}\right)$ modes, and combinations with CCC bending/CC torsion, $v_{24}^{\prime }\left(e_{g}\right)$ mode, with mean energy values of 0.130, 0.095 and 0.051 eV, respectively (Table 2). The Rydberg excitations are assigned to $3p{\left(4e_{u}\right)}^{-1}$ and $3p\prime {\left(4e_{u}\right)}^{-1}$ (see Section 3.4), although this is not in agreement with the character of the calculated electronic transition in Table 1, 3p′/3p′′ 5eu←σCC/CH4eg, 2Eu1←X˜A1g1, and 3p 4a2u←σCH4a1g, 2A2u1←X˜A1g1.

### 3.3. The 8.8–10.8 eV Photon Energy Range

From the theoretical calculations in Table 1, the absorption features in this photon energy region are assigned as mixed valence–Rydberg and Rydberg in character, with vibrational fine structure. Figure 4 shows an expanded view of the photoabsorption spectrum, and the proposed assignments are summarized in Table 4. Most of the Rydberg features appear too broad rather than narrow, and are barely visible in the high energy region of the photoabsorption band. This is due to their energy positions being superimposed on the extensive vibrational progressions of other Rydberg series, spanning from 8.8 up to 10.8 eV, and to the relevant dissociative nature of the $\sigma_{CH}^{*}$ antibonding valence state (see Table 1).

The three dominant electronic states peaking at 9.429, 9.92(7), and 10.393 eV, with cross-section values of 42.53, 66.36, and 86.81 Mb, and calculated oscillator strengths of ≈0.09408, ≈0.10617, and ≈0.17368, respectively, are assigned to nf 5a2u←σCC/CH4eg, 5Eu1←X˜A1g1, σCH∗/3s 5a1g←σCH3eu+nf 6a2u/σCC∗/ns 7a2u←σCC/CH4eg, 8Eu1←X˜A1g1, and np 7a2u←σCH4a1g, 10A2u1←X˜A1g1 (see Table 1). The $0_{0}^{0}$ transitions are tentatively assigned at 8.952, 9.45(7), and 9.73(2) eV, respectively, (Table 4 and Table 5), and show a fine structure superimposed on the Rydberg series (see discussion in Section 3.4). Note that for the first transition, there is also a progression of the CH_2_ rocking, $v_{4}^{\prime }\left(a_{1g}\right)$ mode, as listed in Table 4. The vertical dashed lines in Figure 4 show the tentative assignments of less-resolved features.

### 3.4. Rydberg Transitions

The photoabsorption spectrum above 7.5 eV shows features that have been assigned to different Rydberg transitions, with fine structure in some of the measured photon energy regions (Figure 2, Figure 3 and Figure 4). These Rydberg states converge to ${\left(4e_{u}\right)}^{-1}$ B˜Eu2 and ${\left(3e_{u}\right)}^{-1}$ D˜Eu2 ionic electronic states of cyclohexane and have been assigned in Table 5, according to their positions and the quantum defects obtained from the Rydberg formula: $E_{n}=IE-R/{\left(n-\delta \right)}^{2}$, where *IE* is ionization energy of a given MO, *n* is the principal quantum number of the Rydberg orbital of energy *E*_n_, *R* is the Rydberg constant (13.61 eV), and *δ* is the quantum defect resulting from the penetration of the Rydberg orbital into the core.

A higher uncertainty of features in the spectrum due to increased noise at the highest energies means that only tentative assignments are made above 10.4 eV and are marked as dashed lines in Figure 4. Nonetheless, the lowest-lying Rydberg transition (*n* = 3) converging to the ionic electronic second excited state IE_3_, (4e_u_)^−1^, is assigned to the $\left(3s\leftarrow 4e_{u}\right)$ excitation, with the first member at 7.583 eV and a quantum defect *δ* = 1.00. Higher-order Rydberg members of the ns series up to *n* = 8 are reported in Table 5. The first members of the two $\left(np\leftarrow 4e_{u}\right)$ and $\left(np\prime \leftarrow 4e_{u}\right)$ series display absorption features at 8.41(1) eV and 8.75(0) eV (*δ* = 0.70 and 0.53). In Table 5, we also include an *nd* $\left(nd\leftarrow 4e_{u}\right)$ series with principal quantum numbers up to *n* = 6, where *n* = 3 has been assigned at 9.23(9) eV (*δ* = 0.20). The features at 10.445 eV could also be assigned to 3p(3e_u_)^−1^.

The Rydberg series converging to the ionic electronic fourth excited state IE_5_, (3e_u_)^−1^, are listed in Table 5, and have been assigned to the $\left(ns,\;np,np\prime \leftarrow 3e_{u}\right)$ transitions. The members of these series are only *n* = 3, and are associated with features at 9.611, 10.445, and 10.76(3) eV, with quantum defects *δ* = 1.00, *δ* = 0.70, and *δ* = 0.55, respectively (Table 3). Higher members of these series lie outside the energy range investigated here.

To assess the role of different vibrational modes assigned in the absorption spectrum to the molecular structure of cyclohexane, we have obtained, at the DFT/CAMB3LYP/aug-cc-pVTZ level, the bond lengths in Å and bond angles in (°) for the neutral *chair* ground-state (Figure S1) and the cationic *boat* ground-state (Figure S2). A close comparison between the two molecular structures shows that upon ionization, minor changes up to 3% are noted within the bond lengths. However, relevant shortenings of 10 and 11% are observed within the H11–C3–C4 (also H15–C5–C4 and H17–C6–C1) and H9–C2–C1 angles. These changes are consistent with the general molecular deformation related to CH_2_ rocking, $v_{4}^{\prime }\left(a_{1g}\right)$ and CCC bending/CC torsion $v_{24}^{\prime }\left(e_{g}\right)$ modes. Another interesting aspect is related to a modest 7% decrease in ∡ C5–C4–C3, which may also render a contribution to CH_2_ scissoring, $v_{3}^{\prime }\left(a_{1g}\right),$ and C–C stretching, $v_{5}^{\prime }\left(a_{1g}\right),$ modes, relevant in the vibrational excitation within the Rydberg transitions listed in Table 3 and Table 4 (see also Figure 3 and Figure 4).

### 3.5. Potential Energy Curves for CH_2_ Scissoring and CH_2_ Rocking Coordinates

**Figure 5:**
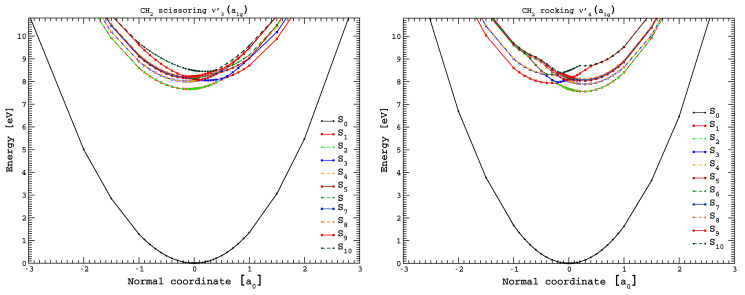
Potential energy curves for the ground and the ten lowest-lying singlet excited states of cyclohexane, following the CH_2_ scissoring, $v_{3}^{\prime }\left(a_{1g}\right),$ and the CH_2_ rocking, $v_{4}^{\prime }\left(a_{1g}\right),$ modes (in a_0_ units). The calculations were performed at the TDDFT/CAMB3LYP/aug-cc-pVTZ level of theory in the *C*_1_ symmetry group. See text for details.

We have obtained potential energy curves (PECs) for the ten lowest excited states of cyclohexane (Figure 5), following the normal coordinates associated with the vibrational modes CH_2_ scissoring, $v_{3}^{\prime }\left(a_{1g}\right),$ and CH_2_ rocking, $v_{4}^{\prime }\left(a_{1g}\right)$ (in a_0_ units). Some of the electronic transitions are not allowed within the *D*_3d_ symmetry point group, i.e., *f*_L_ = 0 in Table S1. However, upon electronic excitation from the ground-state and within the normal coordinate displacement (Figure 5), symmetry is broken. Therefore, the calculations have been performed at the TDDFT/CAMB3LYP/aug-cc-pVTZ level of theory in the *C*_1_ symmetry group, while allowing all atoms to relax following the respective mode.

A close inspection of Figure 5 shows that all states are bound within the reaction coordinates, which is in support of the fine structure involving these modes throughout the different sections of the photoabsorption spectrum. Additionally, the PECs show quasi-degenerate behavior and avoided crossings for most of the calculated electronic states. Thus, within the adiabatic description of the nuclear dynamics, as the normal coordinate is changed, an interchange between states may be possible, i.e., within Rydberg characters. Note that in case of C–C stretching $v_{5}^{\prime }\left(a_{1g}\right)$ mode (see Figure S4), such curve crossing is not relevant, failing to render a relevant vibrational excitation, with only a few quanta being excited. However, the PECs for the doubly degenerate CCC bending/CC torsion, $v_{24}^{\prime }\left(e_{g}\right),$ mode show some avoided crossings, although most of the excited states are strongly bound across a large range of the normal coordinate (see Figure S4). Another relevant aspect of the CCC bending/CC torsion, $v_{24}^{\prime }\left(e_{g}\right),$ mode is the expected Jahn–Teller distortion, which results in a non-totally symmetric displacement of the potential. This is clearly noted for the first lowest-lying excited electronic state in Figure S4 (in red). A close inspection of the figure shows a weak vibronic interaction that is in good agreement with the fine structure mainly noted in the 8.8–10.8 eV photon energy range (Figure 4).

The lowest-lying electronic state in Table 1, 3p 4a2u←σCC/CH4eg, 1Eu1←X˜A1g1, has been calculated at 8.026 eV, ~0.6 eV above the experimental value of 7.46(9) eV. The PECs in Figure 5 for CH_2_ rocking, $v_{4}^{\prime }\left(a_{1g}\right),$ mode show that within the Franck–Condon region, the most probable transition occurs to the quasi-degenerate first and second electronic states at ~7.7 eV. However, if the normal coordinate is slightly reduced to +0.3 a_0_, a vertical excitation energy value of the first excited electronic state in the adiabatic description occurs at 7.5 eV. This change in geometry is in agreement with the energy position of the lowest-lying absorption band (Figure 2). Another relevant aspect is related to the normal coordinate increase to −0.3 a_0_, yielding a vertical excitation energy close to 8 eV. Note that at this position, the potential energy curve is rather shallow, rendering them as Rydberg in character, as obtained from the calculations. The system can now either tunnel through the almost negligible barrier (Figure 5, 3rd excited state in blue), or if the normal mode description is allowed to relax back to the equilibrium position, the excess energy may leave the molecule vibrationally (and rotationally) excited. This is in agreement with the different quanta of the CH_2_ rocking, $v_{4}^{\prime }$ mode, being excited, as noted in the photoabsorption spectrum.

The higher energy states are mostly degenerate at the equilibrium distance, as they originate from the same (4e_g_) and (4a_1g_) molecular orbitals. The intricate molecular dynamics at these energies, and mostly visible for the CH_2_ rocking, $v_{4}^{\prime }$ mode, are also governed by the relevant adiabatic character, mainly at the equilibrium geometry, and as the relative normal coordinate is displaced to +0.5 a_0_ (Figure 5).

### 3.6. Absolute Photoabsorption Cross-Sections and Atmospheric Photolysis

This work reports the absolute photoabsorption cross-sections of cyclohexane in the photon energy region from 6.8 up to 10.8 eV. Table 1 lists the dominant electronic excitations and their values in units of Mb. In comparison, previous studies of the vacuum ultraviolet photoabsorption of C_6_H_12_ include the wavelength regions 124–248 nm (10–5 eV) [24], 154–180 nm (8.059–6.881 eV) [25], 40–120 nm (10.332–30.996 eV) [21], and 127–182 nm (9.733–6.819 eV) [26]. Doner et al. [24] report a cross-section value at 8.5 eV of ≈35 Mb, in good agreement with the present value of 33.96 Mb (at 8.486 eV), while Raymonda’s [26] absolute value yields 16.6 Mb. Pickett et al. [25] report 1.66 Mb at 7.439 eV, a value 28% lower than the 2.28 Mb from this work, while the photoabsorption spectrum of Koizumi et al. [21] report a cross-section of ≈ 90 Mb at 10.332 eV (120 nm), slighter higher than our value of 86.28 Mb.

The radiation reaching the Earth’s atmosphere, which may have an impact on a molecule’s photolysis rate, occurs for wavelengths above 180 nm, and cyclohexane does not show any absorption above this value; therefore, photolysis is not a relevant process. However, gas-phase kinetics for C_6_H_12_ reactions with atmospheric relevant radicals such as ^•^OH and O_3_ have been reported [1,5]. At room temperature, Harley and Cass report a reaction rate of k_OH_ = 7.54 × 10^−12^ cm^3^ molec^−1^ s^−1^ [1] whereas Atkinson recommends a value of 7.21 × 10^−12^ cm^3^ molec^−1^ s^−1^, with an estimated overall uncertainty of ± 20% [5]. Other rate constants at 295 K are reported for gas-phase reactions with ozone, k(O_3_) ≤ 1.3 × 10^−21^ cm^3^ molec^−1^ s^−1^, meaning that the main atmospheric sink mechanism of cyclohexane is the reaction with the ^•^OH radical [5,6].

## 4. Materials and Methods

We have performed vacuum ultraviolet (VUV) photoabsorption experiments in a wide wavelength range from 115 nm up to 182 nm (10.8–6.8 eV). The measurements were carried out using the UV1 beam line on ASTRID, Aarhus University, Denmark, and the specifics of the experiment have previously been described in detail [33,34]. Briefly, the VUV radiation passes through an absorption gas cell end station filled with a static gas sample of cyclohexane vapour at room temperature. The resolution of the photons produced by the monochromator is better than 0.08 nm [33], yielding 1, 3, and 7 meV at the low extreme, the midpoint, and the high extreme of the photon energy range scanned, respectively. The transmitted light is detected by a photomultiplier tube (PMT), and two MgF_2_ transmission windows enclosing the cell set the lower wavelength limit of detection (115 nm). In each absorption scan, the sample’s absolute pressure in the absorption cell is measured by a capacitance manometer (Chell CDG100D, Cambridge, UK), providing the molecular number density needed to obtain the absolute photoabsorption cross-section values, *σ*, in units of megabarns (1Mb ≡ 10^−18^ cm^2^). These are obtained from the Beer–Lambert attenuation law: $I_{t}=I_{0}e^{\left(-N\sigma l\right)}$, where *I_t_* is the light intensity transmitted through the gas sample, *I_o_* is that obtained through the evacuated cell, *N* the molecular number density of cyclohexane, and *l* the absorption path length (15.5 cm). The absolute photoabsorption cross-section values were measured in the pressure range 0.02–1.34 mbar to achieve attenuations of 50% or less, hence avoiding saturation effects.

Accurate cross-section values are obtained by recording the VUV spectrum in small (5 or 10 nm) sections, allowing an overlap of at least 10 points between the adjoining sections and optimizing the pressure used for the measurement, based on the cross-sections of each section, thus allowing us to determine photoabsorption cross-sections to an accuracy of ±5%.

The liquid sample of cyclohexane (CAS number: 110-82-7) used in the VUV photoabsorption measurements was purchased from Sigma-Aldrich (St. Gallen, Switzerland), with a stated purity of ≥99.5%. The sample was degassed through repeated freeze-pump-thaw cycles before use.

Quantum chemical calculations were completed at the DFT/TDDFT [35,36] level, with a CAMB3LYP functional [37], and the aug-cc-pVTZ basis set as implemented in the GAMESS-US computational package (version GAMESS-30 Jun 2020 (R1)) [38]. Initially, the calculations were performed at the DFT level, where we optimized the geometry of all possible conformations of cyclohexane and calculated the harmonic frequencies, verifying the stability of each conformer, as well as the total energy plus ZPE to calculate the Boltzmann distribution. Furthermore, we applied the same methodology to the cationic ground-state. For the neutral molecule, the *chair* and the *twist-boat* conformers were stable. However, the latter is 0.27 eV higher in energy than the former, and therefore, it is not expected to be relevant at room temperature (Table S1); as far as the cation is concerned, its electronic ground-state geometry is the *boat* conformation (Section 2), with bond lengths in Å and bond angles in (°), as shown in Figure S2. TDDFT calculations have been performed to compute the vertical excitation energies and oscillator strengths to aid in the assignment of the VUV absorption spectrum. In Table 1, we list the most dominant vertical excitation energies and oscillator strengths of the electronically excited states, together with the experimental values and related cross-section values. Cyclohexane belongs to the *D*_3d_ symmetry point group, but the calculations have been performed using the Abelian sub-group *C*_2h_. In the Supplementary Table S2, a complete set of the calculated electronic transitions can be found, together with the oscillator strengths. Additionally, harmonic frequencies at the DFT-CAMB3LYP/aug-cc-pVTZ level for the neutral and the cation electronic ground-states have been calculated (Tables S3 and S4) and used to assign the major vibrational features within the mixed valence–Rydberg and Rydberg excitations in the VUV spectrum (Figure 1, Figure 2, Figure 3 and Figure 4). To further our knowledge regarding the molecular structure of C_6_H_12_, we have also computed the optimized neutral and cationic ground-states, while checking all possible conformations. For the neutral molecule, the *chair* and the *twist-boat* conformers were obtained; however, the latter is 0.267 eV higher in energy than the former, and as such, it is not expected to be relevant at room temperature (Table S4); as far as the cation is concerned, its electronic ground-state geometry is the *boat* (Section 2, Figures S1 and S2).

## 5. Conclusions

In this joint experimental and theoretical work regarding the electronic state spectroscopy of cyclohexane, mixed valence–Rydberg and Rydberg character electronic excitations have been assigned in the energy range 6.8–10.8 eV (182–115 nm). The calculations at the DFT/CAMB3LYP/aug-cc-pVTZ level have provided vertical excitation energies and oscillator strengths for the major electronic transitions. The fine structure assignment involves vibronic excitations, with the contribution of the CH_2_ scissoring, $v_{3}^{\prime }\left(a_{1g}\right)$, the CH_2_ rocking, $v_{4}^{\prime }\left(a_{1g}\right)$, the C–C stretching, $v_{5}^{\prime }\left(a_{1g}\right),$ and the CCC bending/CC torsion, $v_{24}^{\prime }\left(e_{g}\right),$ modes.

The photolysis of cyclohexane does not play a role in the Earth’s atmosphere from 0 km altitude up to 50 km (the limit of the stratopause), while gas-phase kinetic studies of C_6_H_12_ reactions with the ^•^OH radical have previously been shown to be the prevalent atmospheric sink mechanism [1,5]. Time-dependent density functional theory, with the aug-cc-pVTZ basis set, has been used to calculate potential energy curves for the ten lowest-lying singlet excited states of cyclohexane along the CH_2_ scissoring, $v_{3}^{\prime }\left(a_{1g}\right),$ and the CH_2_ rocking, $v_{4}^{\prime }\left(a_{1g}\right),$ coordinates, mainly contributing to the spectrum yielding a relevant interchange character between Rydberg states. Other PECs have also been obtained for C–C stretching $v_{5}^{\prime }\left(a_{1g}\right)$ and CCC bending/CC torsion $v_{24}^{\prime }\left(e_{g}\right)$ modes. The results of the rather complex nuclear dynamics involving the contribution of these modes show the need to map the potential energy surfaces and the role of possible conical intersections, which would require a computational expense that is beyond the scope of the present work.

## Figures and Tables

**Table 1:** The dominant calculated vertical excitation energies and oscillator strengths (TDDFT/CAMB3LYP/aug-cc-pVTZ) of cyclohexane compared with the present experimental data (energies in eV).

Cyclohexane					E (eV) Expt. ^2^	Cross- Section (Mb)
State (*D*_3d_)	State (*C*_2h_)	E (eV)	*f*_L_	Dominant Excitations ^1^		
X˜A1g1	X˜Ag1					
1 ^1^E_u_	$A_{u}\;⊕$ B_u_	8.026	0.000172	3p(4a_2u_) ← $\sigma_{CC/CH}$(4e_g_) (90%)	7.46(9)	3.49
1 ^1^A_2u_	B_u_	8.147	0.002014	3p′(5e_u_) ← $\sigma_{CC/CH}$(4e_g_) (44%), 3p″(5e_u_) ← $\sigma_{CC/CH}$(4e_g_) (44%)	8.02(0)	17.99
2 ^1^E_u_	$A_{u}\;⊕$ B_u_	8.198	0.034207	${3p}^{\prime }$(5e_u_) ← $\sigma_{CC/CH}$(4e_g_) (43%), 3p″(5e_u_) ← $\sigma_{CC/CH}$(4e_g_) (43%)	8.07(7)	18.57
2 ^1^A_2u_	B_u_	8.487	0.221001	3p(4a_2u_) ← $\sigma_{CH}$(4a_1g_) (90%)	8.486	33.96
5 ^1^E_u_	$A_{u}\;⊕$ B_u_	9.677	0.094079	nf(5a_2u_) ← $\sigma_{CC/CH}$(4e_g_) (83%)	9.429	42.53
8 ^1^E_u_	$A_{u}\;⊕$ B_u_	10.014	0.106166	$\sigma_{CH}^{*}/3s$(5a_1g_) ← $\sigma_{CH}$(3e_u_) (28%), nf(6a_2u_) ← $\sigma_{CC/CH}$(4e_g_) (33%), $\sigma_{CC}^{*}/ns$(7a_2u_) ← $\sigma_{CC/CH}$(4e_g_) (13%)	9.92(7)	66.36
10 ^1^A_2u_	B_u_	10.751	0.173676	np(7a_2u_) ← $\sigma_{CH}$(4a_1g_) (87%)	10.393	86.81

^1^ We are not able to give the principal quantum number for the Rydberg members assigned with *n*. ^2^ The last decimal of the energy value is given in brackets for these less-resolved features.

**Table 2:** Proposed vibrational assignments of cyclohexane absorption bands in the photon energy range of 6.9−8.0 eV ^a^; energies in eV.

Assignment	Energy	ΔE (*ν*_3_′)	ΔE (*ν*_4_′)	ΔE (*ν*_5_′)
3p 4a2u←σCC/CH4eg, 1Eu1←X˜A1g1				
$0_{0}^{0}$	7.085	–	–	–
$p_{1}4_{0}^{0}$	7.105	–	–	–
$p_{2}4_{0}^{0}$	7.130	–	–	–
$p_{3}4_{0}^{0}$	7.146	–	–	–
$p_{4}4_{0}^{0}$	7.154	–	–	–
$p_{5}4_{0}^{0}/5_{0}^{1}$	7.17(9) (s,w)	–	–	0.094
$4_{0}^{1}/p_{1}5_{0}^{1}$	7.20(0) (s)	–	0.115	0.095
$p_{1}4_{0}^{1}/p_{2}4_{0}^{0}5_{0}^{1}$	7.22(1) (w)	–	0.116	0.091
$p_{2}4_{0}^{1}3_{0}^{1}$	7.238	0.153	0.108	–
$p_{3}4_{0}^{1}/p_{4}5_{0}^{1}$	7.26(3) (s)	–	0.117	0.109
$p_{4}4_{0}^{1}/p_{2}3_{0}^{1}/p_{5}4_{0}^{0}5_{0}^{1}/5_{0}^{2}$	7.276	0.146	0.122	0.097
$p_{5}4_{0}^{1}$	7.28(9) (w)	–	0.110	–
$4_{0}^{2}/p_{2}4_{0}^{0}5_{0}^{2}$	7.31(9) (s)	–	0.119	0.098
$p_{1}4_{0}^{2}$	7.32(8) (s)	–	0.107	0.090
$p_{2}4_{0}^{2}$	7.345	–	0.107	–
$p_{3}4_{0}^{2}/p_{4}4_{0}^{1}5_{0}^{1}/p_{2}3_{0}^{1}5_{0}^{1}/p_{5}4_{0}^{0}5_{0}^{2}/5_{0}^{3}$	7.38(0) (s)	–	0.117	0.104
$p_{4}4_{0}^{2}/p_{2}3_{0}^{2}$	7.389	0.151	0.113	–
$p_{5}4_{0}^{2}/4_{0}^{2}5_{0}^{1}/p_{2}4_{0}^{0}5_{0}^{3}$	7.41(5) (s)	–	0.126	0.096
$p_{4}4_{0}^{1}3_{0}^{1}/p_{2}3_{0}^{2}/p_{5}4_{0}^{0}5_{0}^{1}3_{0}^{1}/5_{0}^{2}3_{0}^{1}/p_{1}4_{0}^{2}5_{0}^{1}$	7.42(9) (s,w)	0.153	–	0.101
$4_{0}^{3}$	7.43(3) (s)	–	0.114	–
$p_{1}4_{0}^{3}$	7.44(2) (s)	–	0.114	–
$p_{2}4_{0}^{3}$	7.44(7) (s)	–	0.102	–
$p_{4}4_{0}^{3}/p_{2}3_{0}^{2}4_{0}^{1}/p_{5}4_{0}^{2}5_{0}^{1}/4_{0}^{2}5_{0}^{2}/p_{2}4_{0}^{0}5_{0}^{4}$	7.51(4) (s,b)	–	0.125	0.099
$p_{5}4_{0}^{3}/p_{4}4_{0}^{1}3_{0}^{1}5_{0}^{1}/p_{2}3_{0}^{2}5_{0}^{1}/p_{5}4_{0}^{0}5_{0}^{2}3_{0}^{1}/5_{0}^{3}3_{0}^{1}/p_{1}4_{0}^{2}5_{0}^{2}$	7.52(8) (b,w)	–	0.113	0.099
$4_{0}^{4}/p_{4}4_{0}^{2}3_{0}^{1}/p_{2}3_{0}^{3}$	7.54(6) (s)	0.157	0.113	–
$p_{2}4_{0}^{4}$	7.56(0) (s)	–	0.113	–
$p_{4}4_{0}^{1}3_{0}^{2}/p_{2}3_{0}^{3}/p_{5}4_{0}^{0}5_{0}^{1}3_{0}^{2}/5_{0}^{2}3_{0}^{2}/p_{1}4_{0}^{2}5_{0}^{1}3_{0}^{1}$	7.57(0) (s)	0.141	–	–
$p_{4}4_{0}^{4}/p_{2}3_{0}^{2}4_{0}^{2}/p_{5}4_{0}^{3}5_{0}^{1}/p_{4}4_{0}^{1}3_{0}^{1}5_{0}^{2}/p_{2}3_{0}^{2}5_{0}^{2}/p_{5}4_{0}^{0}5_{0}^{3}3_{0}^{1}/5_{0}^{4}3_{0}^{1}/p_{1}4_{0}^{2}5_{0}^{3}$	7.625	–	0.111	0.097
$4_{0}^{5}$	7.67(2) (s)	–	0.126	–
$p_{2}4_{0}^{5}$	7.67(7) (s)	–	0.117	–
$4_{0}^{4}3_{0}^{1}/p_{4}4_{0}^{2}3_{0}^{2}/p_{2}3_{0}^{4}$	7.69(6) (s,b)	0.150	–	–
$4_{0}^{4}3_{0}^{2}/p_{4}4_{0}^{2}3_{0}^{3}/p_{2}3_{0}^{5}$	7.84(7) (s,w)	0.151	–	–
	$\bar{\Delta E}$	0.150	0.115	0.098
3p/3p′ 5eu←σCC/CH4eg, 1A2u1←X˜A1g1				
$0_{0}^{0}$	7.345	–	–	–
$4_{0}^{1}$	7.46(9)	–	0.124	–
$3_{0}^{1}$	7.51(0) (w)	0.165	–	–
$4_{0}^{2}$	7.583	–	0.114	–
$3_{0}^{2}$	7.67(7) (s)	0.167	–	–
$4_{0}^{3}$	7.69(6) (s,b)	–	0.113	–
$4_{0}^{4}$	7.82(2) (s,b)	–	0.126	–
$4_{0}^{5}$	7.94(3) (s,b)	–	0.121	–
	$\bar{\Delta E}$	0.153	0.120	–

^a^ (w) weak feature; (s) shoulder structure; (b) broad feature (the last decimal of the energy value is given in brackets for these less-resolved features).

**Table 3:** Proposed vibrational assignments of cyclohexane absorption bands in the photon energy range of 7.8−8.8 eV ^a^; energies in eV.

Assignment	Energy	ΔE (*ν*_4_′)	ΔE (*ν*_5_′)	ΔE (*ν*_24_′)
3p′/3p″ 5eu←σCC/CH4eg, 2Eu1←X˜A1g1				
$0_{0}^{0}$	7.80(8) (s)	–	–	–
$5_{0}^{1}$	7.89(2) (s)	–	0.084	–
$4_{0}^{1}$	7.93(3) (s)	0.125	–	–
$5_{0}^{1}4_{0}^{1}$	8.02(0) (s)	0.128	–	–
$4_{0}^{2}$	8.06(7) (s)	0.134	–	–
$4_{0}^{3}$	8.19(5) (s,w)	0.128	–	–
3p 4a2u←σCH4a1g, 2A2u1←X˜A1g1				
$0_{0}^{0}$	8.22(7) (s)	–	–	–
$24_{0}^{1}$	8.27(7) (s)	–	–	0.050
$5_{0}^{1}$	8.32(7) (s,w)	–	0.100	–
$4_{0}^{1}$	8.36(0) (s,w)	0.133	–	–
$4_{0}^{1}5_{0}^{1}$	8.41(1) (s,b)	0.134	–	0.051
$4_{0}^{1}24_{0}^{1}$	8.46(3) (s,w)	0.136	–	–
$4_{0}^{2}$	8.486	0.126	–	–
$4_{0}^{2}5_{0}^{1}$	8.53(9) (s,w)	0.128	–	0.053
$4_{0}^{2}24_{0}^{1}$	8.58(6) (w)	0.123	0.100	–
$4_{0}^{3}$	8.62(2) (b)	0.136	–	
$4_{0}^{3}5_{0}^{1}$	8.67(0) (b,w)	0.131	–	0.048
$4_{0}^{3}24_{0}^{1}$	8.71(9) (w)	0.133	0.097	–
$4_{0}^{4}$	8.75(0) (b,w)	0.128	–	–
	$\bar{\Delta E}$	0.130	0.095	0.051

^a^ (s) shoulder structure; (w) weak feature; (b) broad feature (the last decimal of the energy value is given in brackets for these less-resolved features).

**Table 4:** Proposed vibrational assignments and progression of cyclohexane absorption bands in the photon energy range of 8.8−10.8 eV ^a^; energies in eV.

Assignment	Energy	ΔE (*ν*_3_′)	ΔE (*ν*_4_′)	ΔE (*ν*_5_′)	ΔE (*ν*_24_′)
nf 5a2u←σCC/CH4eg, 5Eu1←X˜A1g1					
$p_{1}4_{0}^{0}$	8.93(3) (s)	–	–	–	–
$0_{0}^{0}$	8.952	–	–	–	–
$5_{0}^{1}$	9.03(7) (w)	–	–	0.084	–
$p_{1}4_{0}^{1}$	9.06(3) (s)	–	0.130	–	–
$4_{0}^{1}$	9.083	–	0.131	–	–
$3_{0}^{1}$	9.110	0.158	–	–	–
$4_{0}^{1}5_{0}^{1}$	9.17(0) (b)	–	0.133	0.087	–
$p_{1}4_{0}^{2}$	9.205	–	0.142	–	–
$4_{0}^{2}$	9.23(2) (b)	–	0.149	–	–
$4_{0}^{2}24_{0}^{1}$	9.294	–	0.124	–	0.062
$p_{1}4_{0}^{3}$	9.35(0) (s)	–	0.145	–	–
$4_{0}^{3}$	9.37(1) (w)	–	0.139	–	–
$4_{0}^{3}24_{0}^{1}$	9.429	–	0.135	–	0.058
$p_{1}4_{0}^{4}$	9.479	–	0.129	–	–
$4_{0}^{4}24_{0}^{1}$	9.559	–	0.130	–	–
$p_{1}4_{0}^{5}$	9.611	–	0.132	–	–
$4_{0}^{5}24_{0}^{1}$	9.69(4) (s,w)	–	0.135	–	–
$p_{1}4_{0}^{6}$	9.74(7) (w)	–	0.129	–	–
σCH∗/3s 5a1g←σCH3eu+nf 6a2u/σCC∗/ns 7a2u←σCC/CH4eg, 8Eu1←X˜A1g1					
$0_{0}^{0}$	9.45(7) (w)	–	–	–	–
$4_{0}^{1}$	9.589	–	0.132	–	–
$3_{0}^{1}$	9.611	0.154	–	–	–
$4_{0}^{2}$	9.72(4) (w)	–	0.135	–	–
$3_{0}^{2}$	9.77(8) (s,b)	0.167	–	–	–
$4_{0}^{3}$	9.86(4) (s,w)	–	0.140	–	–
$3_{0}^{3}$	9.92(7) (s,w)	0.149	–	–	–
$4_{0}^{4}$	9.99(9) (s,w)	–	0.135	–	–
$4_{0}^{5}$	10.14(6) (s,w)	–	0.147	–	–
$4_{0}^{6}$	10.28(1) (s,w)	–	0.135	–	–
$4_{0}^{7}$	10.41(0) (s,w)	–	0.129	–	–
$4_{0}^{8}$	10.55(2) (s,w) *	–	0.142	–	–
$4_{0}^{9}$	10.69(8) (s,w) *	–	0.146	–	–
	$\bar{\Delta E}$	0.157	0.136	0.086	0.060

^a^ (s) shoulder structure; (w) weak feature; (b) broad feature; * tentative assignment (the last decimal of the energy value is given in brackets for these less-resolved features).

**Table 5:** Energy values (eV), quantum defects (*δ*), and assignments of the Rydberg series converging to the 4eu−1 B˜Eu2 and 3eu−1 D˜Eu2 ionic electronic states of cyclohexane ^a^.

*E*_n_	*δ*	Assignment	*E*_n_	*δ*	Assignment
(IE_3_)_v_ = 10.98 eV ${\left(4e_{u}\right)}^{-1}$			(IE_5_)_v_ = 13.03 eV ${\left(3e_{u}\right)}^{-1}$		
(*ns* ← *4e_u_*)			(*ns* ← *3e_u_*)		
7.583	1.00	3s	9.611	1.00	3s
9.479	0.99	4s	–	–	–
10.14(6) (s,w)	0.96	5s	–	–	–
10.445	0.96	6s *	–	–	–
10.60(6) (w)	0.97	7s *	–	–	–
10.69(8) (w)	1.05	8s *	–	–	–
(*np* ← *4e_u_*)			(*np* ← *3e_u_*)		
8.41(1) (s,b)	0.70	3p	10.445	0.70	3p *
9.73(2) (s)	0.70	4p	–	–	–
10.23(4) (s,w)	0.73	5p	–	–	–
(*np′* ← *4e_u_*)			(*np′* ← *3e_u_*)		
8.75(0) (w)	0.53	3p′	10.76(3) (b,w)	0.55	3p′ *
9.84(8) (s)	0.53	4p′	–	–	–
10.29(8) (s,w)	0.53	5p′	–	–	–
10.52(5) (w)	0.53	6p′ *	–	–	–
10.66(1) (s,w)	0.47	7p′ *	–	–	–
(*nd* ← *4e_u_*)			(*nd* ← *3e_u_*)		
9.23(9) (b,w)	0.20	3d	–	–	–
10.03(9) (s)	0.20	4d	–	–	–
10.38(4) (w)	0.22	5d	–	–	–
10.57(9) (w)	0.17	6d *	–	–	–

^a^ (s) shoulder structure; (w) weak feature; (b) broad structure; * tentative assignment (the last decimal of the energy value is given in brackets for these less-resolved features).

## Data Availability

Data presented in this publication are available upon request from the authors.

## Supplementary Materials

The following supporting information can be downloaded at: https://www.mdpi.com/article/10.3390/molecules30071493/s1, Figure S1: Electronic configuration, Cartesian coordinates, and neutral *chair* ground-state geometry of cyclohexane obtained at the DFT/CAMB3LYP/aug-cc-pVTZ level of theory. Bond lengths are in Å, and bond angles are in (°). The plot was obtained with MacMolPlt graphical interface [39]; Figure S2: Cartesian coordinates and cationic *boat* ground-state geometry cyclohexane obtained at the DFT/CAMB3LYP/aug-cc-pVTZ level of theory. Bond lengths are in Å, and bond angles are in (°). The plot was obtained with MacMolPlt graphical interface [39]; Figure S3: Representation of a selection of cyclohexane molecular orbitals computed at the DFT/CAMB3LYP/aug-cc-pVTZ level of theory; Figure S4: Potential energy curves for the ground- and the ten lowest-lying singlet excited states of cyclohexane, following the CCC bending/CC torsion, $v_{24}^{\prime }\left(e_{g}\right),$ and for C–C stretching, $v_{27/28}^{\prime }\left(e_{g}\right),$ modes (in a_0_ units). The calculations were performed at the TDDFT/CAMB3LYP/aug-cc-pVTZ level of theory in the *C*_1_ symmetry group; Table S1: Total energy values with zero-point correction computed at the DFT/CAMB3LYP/aug-cc-pVTZ level of theory for cyclohexane conformers in the neutral ground-state and first excited state. Also included is the energy difference ΔE with respect to the energy of the most stable conformer; Table S2: The calculated vertical excitation energies and oscillator strengths (TD-DFT/CAMB3LYP/aug-cc-pVTZ) of *chair* cyclohexane (energies in eV). See text for details; Table S3. Harmonic frequencies computed at the DFT/CAMB3LYP/aug-cc-pVTZ level of theory for *chair* cyclohexane neutral electronic ground-state, compared with experimental data [31,40]; Table S4. Harmonic frequencies computed at the DFT/CAMB3LYP/aug-cc-pVTZ level of theory for cationic *boat-axial* ground-state cyclohexane [40].
