# Supplementary material for: Cyclohexane Vibronic States: A Combined VUV Spectroscopy and Theoretical Study
Source: Molecules. 2025 Mar 27;30(7):1493. doi: 10.3390/molecules30071493 (PMC11990123; doi:10.3390/molecules30071493)
Supplement: Supplementary file 1 [file molecules-30-01493-s001.zip › molecules-3544878-supplementary.pdf]

Supplementary Materials

Cyclohexane Vibronic States: A Combined VUV Spectroscopy and Theoretical Study

Edvaldo Bandeira [1], Alessandra S. Barbosa [1], Nykola C. Jones [2], Søren V. Hoffmann [2], Márcio H. F. Bettega [1,*] and Paulo Limão-Vieira [1,3,*]

[1] Departamento de Física, Universidade Federal do Paraná, Caixa Postal 19044, Curitiba 81531-980, PR, Brazil
[2] ISA, Department of Physics and Astronomy, Aarhus University, Ny Munkegade 120, DK-8000 Aarhus C, Denmark
[3] Atomic and Molecular Collisions Laboratory, Centre of Physics and Technological Research (CEFITEC), Department of Physics, NOVA School of Science and Technology, Universidade NOVA de Lisboa, 2829-516 Caparica, Portugal
* Correspondence: bettega@fisica.ufpr.br (M.H.F.B.); plimaovieira@fct.unl.pt (P.L.-V.); Tel.: +55-41-3361-3002 (M.H.F.B.); +351-21-294-78-59 (P.L.-V.)

Figure captions

Figure S1. Electronic configuration, cartesian coordinates and neutral *chair* ground-state geometry of cyclohexane obtained at the DFT/CAMB3LYP/aug-cc-pVTZ level of theory. Bond lengths are in Å and bond angles in (°). The plot was obtained with MacMolPlt graphical interface [1].

Figure S2. Cartesian coordinates and cationic *boat* ground-state geometry cyclohexane obtained at the DFT/CAMB3LYP/aug-cc-pVTZ level of theory. Bond lengths are in Å and bond angles in (°). The plot was obtained with MacMolPlt graphical interface [1].

Figure S3. Representation of a selection of cyclohexane molecular orbitals computed at the DFT/CAMB3LYP/aug-cc-pVTZ level of theory. The plots were obtained with MacMolPlt graphical interface [1].

Figure S4. Potential energy curves for the ground and the ten lowest-lying singlet excited states of cyclohexane, following the CCC bending/CC torsion $v'_{24}(e_g)$ and for C–C stretching, $v'_{27/28}(e_g)$ modes (in a_0 units). The calculations were performed at the TD-DFT/CAMB3LYP/aug-cc-pVTZ level of theory in the C_1 symmetry group. See text for details.

Table caption

Table S1. Total energy values with zero-point correction computed at the DFT/CAMB3LYP/ aug-cc-pVTZ level of theory for cyclohexane conformers in the neutral ground-state and first excited state. Also included is the energy difference ΔE with respect to the energy of the most stable conformer.

Table S2. The calculated vertical excitation energies and oscillator strengths (TD-DFT/CAMB3LYP/aug-cc-pVTZ) of *chair* cyclohexane (energies in eV). See text for details.

Table S3. Harmonic frequencies computed at the DFT/CAMB3LYP/ aug-cc-pVTZ level of theory for *chair* cyclohexane neutral electronic ground-state, compared with experimental data.

Table S4. Harmonic frequencies computed at the DFT/CAMB3LYP/aug-cc-pVTZ level of theory for cationic *boat-axial* ground-state cyclohexane.

Figure S1. Electronic configuration, cartesian coordinates and neutral *chair* ground-state geometry of cyclohexane obtained at the DFT/CAMB3LYP/aug-cc-pVTZ level of theory. Bond lengths are in Å and bond angles in (°). The plot was obtained with MacMolPlt graphical interface [1].

Electronic configuration of $\tilde{X}\ ^1A_{1g}$ state:

core orbitals: $(1a_{1g})^2$ $(1e_u)^4$ $(1e_g)^4$ $(1a_{2u})^2$ $(2a_{1g})^2$ $(2e_u)^4$ $(2e_g)^4$

valence orbitals: $(2a_{2u})^2$ $(3a_{1g})^2$ $(3a_{2u})^2$ $(3e_g)^4$ $(3e_u)^4$ $(1a_{1u})^2$ $(4e_u)^4$ $(4a_{1g})^2$ $(4e_g)^4$

unoccupied orbitals: $(5a_{1g})$ $(4a_{2u})$ $(5e_u)$ $(5e_g)$ $(6a_{1g})$ $(6e_g)$ $(5a_{2u})$ $(6e_u)$ $(7e_u)$ $(6a_{2u})$ $(7a_{1g})$ $(2a_{1u})$ $(7a_{2u})$ $(8e_u)$ $(8e_g)$ $(8a_{1g})$

Cartesian coordinates of all atoms				
Atom	*x* (Å)	*y* (Å)	*z* (Å)	
C1	1.26040208	0.7276935	0.22866955	
C2	1.26040208	-0.7276935	-0.22866955	
C3	0	-1.455387	0.22866955	
C4	-1.26040208	-0.7276935	-0.22866955	
C5	-1.26040208	0.7276935	0.22866955	
C6	0	1.455387	-0.22866955	
H7	2.14908433	1.24077439	-0.14190041	
H8	1.31544864	0.75947464	1.32092595	
H9	2.14908433	-1.24077439	0.14190041	
H10	1.31544864	-0.75947464	-1.32092595	
H11	0	-2.48154879	-0.14190041	
H12	0	-1.51894927	1.32092595	
H13	-2.14908433	-1.24077439	0.14190041	
H14	-1.31544864	-0.75947464	-1.32092595	
H15	-2.14908433	1.24077439	-0.14190041	
H16	-1.31544864	0.75947464	1.32092595	
H17	0	2.48154879	0.14190041	
H18	0	1.51894927	-1.32092595	

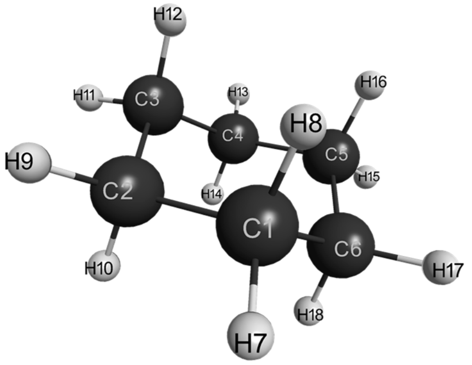

bond length (Å)		angle (°)	
C1 – C2	1.5256	C3 – C2 – C1	111.42
C2 – C3	1.5256	C4 – C3 – C2	111.42
C3 – C4	1.5256	C5 – C4 – C3	111.42
C4 – C5	1.5256	C6 – C5 – C4	111.42
C5 – C6	1.5256	H7 – C1 – C2	110.29
H7 – C1	1.0910	H8 – C1 – H7	106.53
H8 – C1	1.0941	H9 – C2 – C1	110.29
H9 – C2	1.0910	H10 – C2 – H9	106.53
H10 – C2	1.0941	H11 – C3 – C2	110.29
H11 – C3	1.0910	H12 – C3 – H11	106.53
H12 – C3	1.0941	H13 – C4 – C3	110.29
H13 – C4	1.0910	H14 – C4 – H13	106.53
H14 – C4	1.0941	H15 – C5 – C4	110.29
H15 – C5	1.0910	H16 – C5 – H15	106.53
H16 – C5	1.0941	H17 – C6 – C1	110.29
H17 – C6	1.0910	H18 – C6 – H17	106.53
H18 – C6	1.0941		

Figure S2. Cartesian coordinates and cationic *boat-axial* ground-state geometry cyclohexane obtained at the DFT/CAMB3LYP/aug-cc-pVTZ level of theory. Bond lengths are in Å and bond angles in (°). The plot was obtained with MacMolPlt graphical interface [1].

Cartesian coordinates of all atoms			
Atom	*x* (Å)	*y* (Å)	*z* (Å)
C1	-1.37436247	0.00000183	0.41120121
C2	-0.74503022	-1.22804642	-0.32027557
C3	0.74501783	-1.22804976	-0.32024953
C4	1.37437475	0.00000046	0.41118026
C5	0.74501675	1.22804832	-0.32024664
C6	-0.74503195	1.22804654	-0.32027662
H7	-1.12838876	0.00000006	1.46799088
H8	-2.45441985	-0.00000055	0.30618018
H9	-1.1488601	-2.04769325	0.30080441
H10	-1.1645999	-1.32290149	-1.31751633
H11	1.14886093	-2.04770756	0.3007926
H12	1.16460061	-1.32290125	-1.31749403
H13	1.12839758	-0.00000061	1.46797955
H14	2.45442939	-0.00000207	0.3061839
H15	1.14886069	2.04770851	0.30079159
H16	1.16459584	1.32289934	-1.31749272
H17	-1.14886069	2.04769421	0.30080423
H18	-1.16460037	1.32290339	-1.31751728

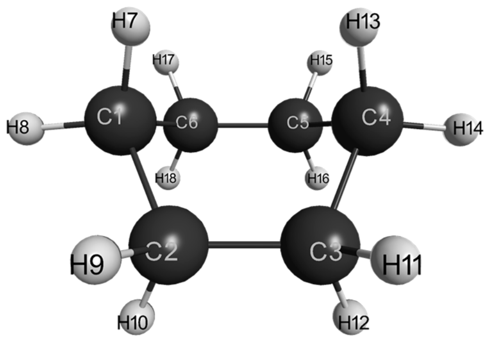

bond length (Å)		angle (°)	
C1 – C2	1.5618	C3 – C2 – C1	113.76
C2 – C3	1.4901	C4 – C3 – C2	113.77
C3 – C4	1.5618	C5 – C4 – C3	103.68
C4 – C5	1.5618	C6 – C5 – C4	113.77
C5 – C6	1.4901	H7 – C1 – C2	111.40
H7 – C1	1.0850	H8 – C1 – H7	108.66
H8 – C1	1.0852	H9 – C2 – C1	99.95

H9 – C2	1.1048	H10 – C2 – H9	108.07
H10 – C2	1.0861	H11 – C3 – C4	99.95
H11 – C3	1.1048	H12 – C3 – H11	108.07
H12 – C3	1.0861	H13 – C4 – C5	111.39
H13 – C4	1.0851	H14 – C4 – H13	108.66
H14 – C4	1.0852	H15 – C5 – C4	99.95
H15 – C5	1.1048	H16 – C5 – H15	108.07
H16 – C5	1.0861	H17 – C6 – C1	99.95
H17 – C6	1.1048	H18 – C6 – H17	108.07
H18 – C6	1.0861		

Figure S3. Representation of a selection of cyclohexane molecular orbitals computed at the DFT/CAMB3LYP/aug-cc-pVTZ level of theory. The plots were obtained with MacMolPlt graphical interface [1].

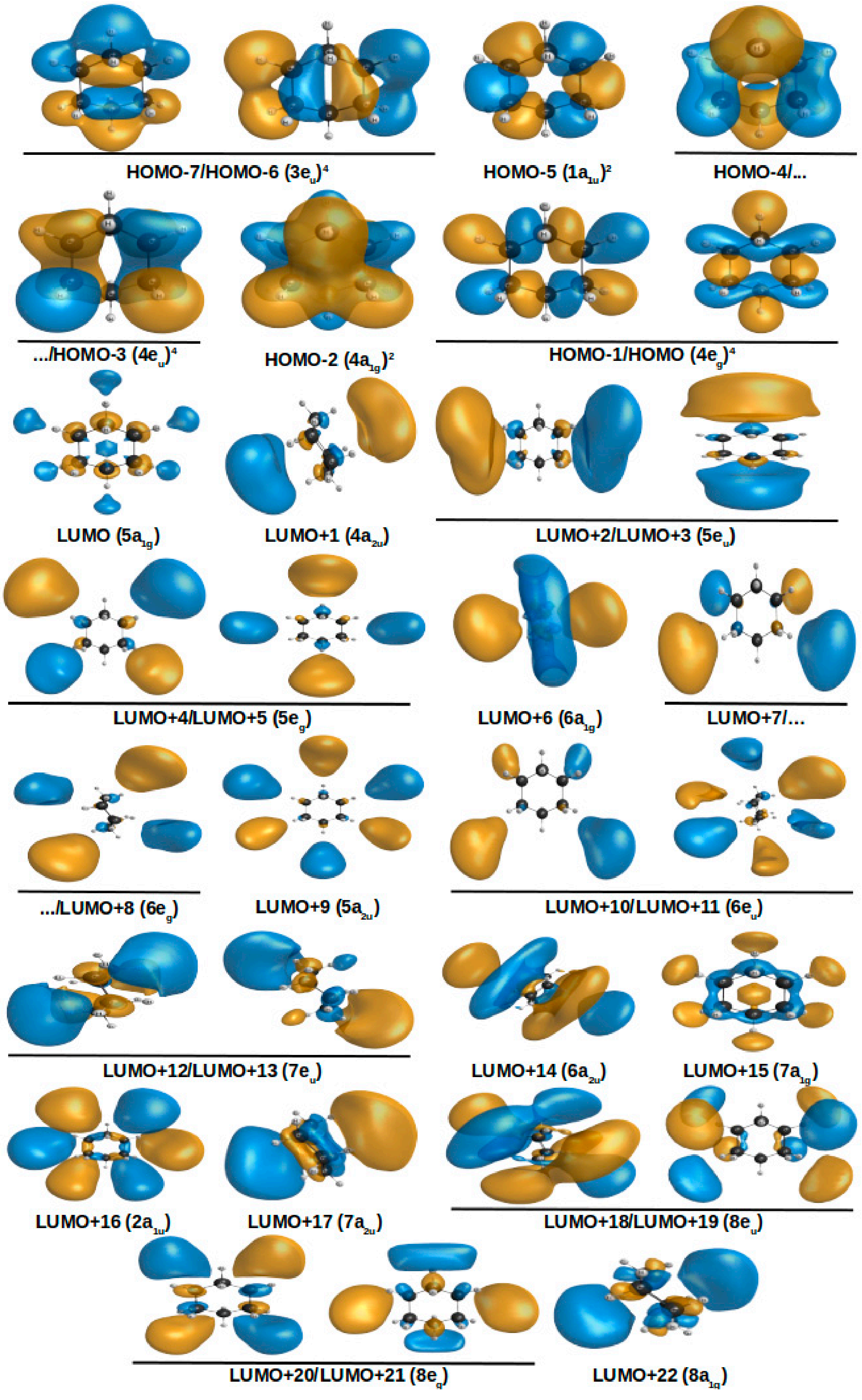

Figure S4. Potential energy curves for the ground and the ten lowest-lying singlet excited states of cyclohexane, following the C–C stretching, $\nu_5'(a_{1g})$ and CCC bending/CC torsion $\nu_{24}'(e_g)$ modes (in a_0 units). The calculations were performed at the TD-DFT/CAMB3LYP/aug-cc-pVTZ level of theory in the C_1 symmetry group. See text for details.

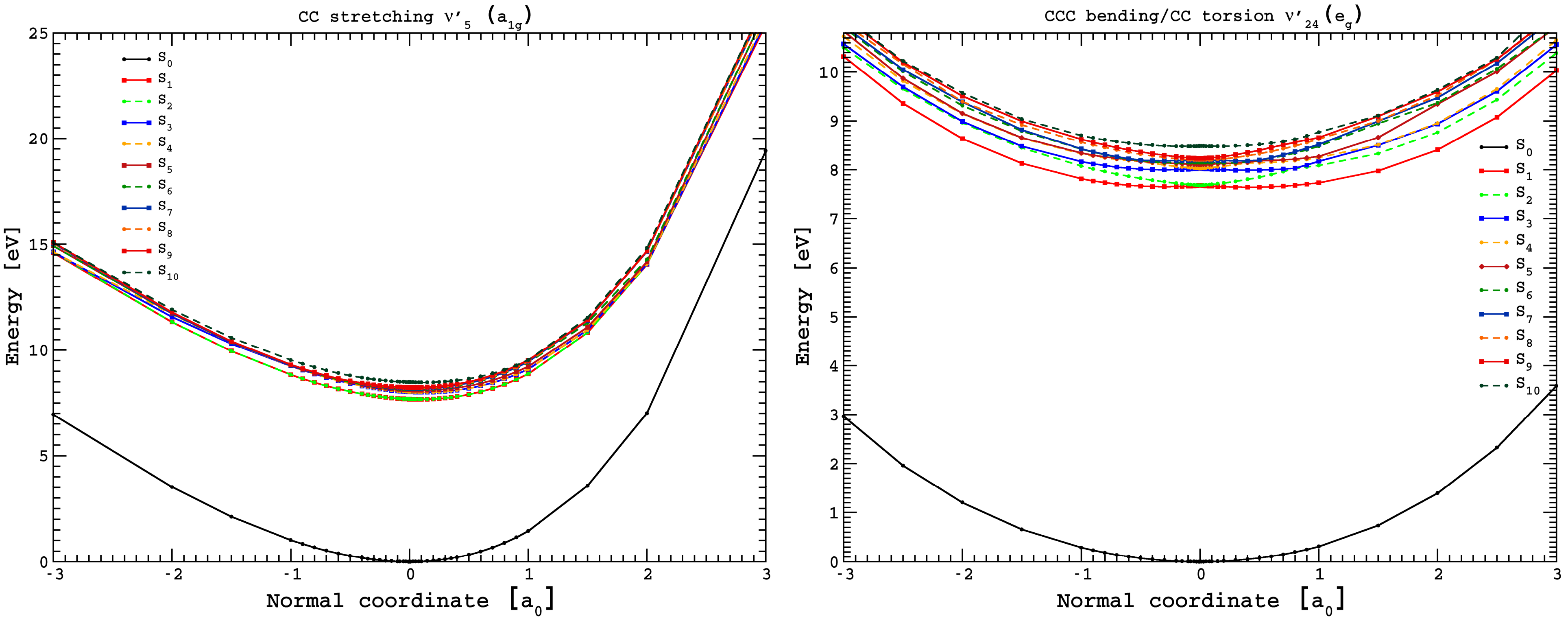

Table S1. Total energy values with zero-point correction computed at the DFT/CAMB3LYP/aug-cc-pVTZ level of theory for cyclohexane conformers in the neutral ground-state and first excited state. Also included is the energy difference ΔE with respect to the energy of the most stable conformer.

$\tilde{X}\ ^1A_{1g}$		
conformer	Energy (Hartree)	ΔE (eV)
chair	-235.644497	–
twist-boat	-235.634694	0.27
$\tilde{X}\ ^2E_g$		
boat-axial	-235.2582303	10.51

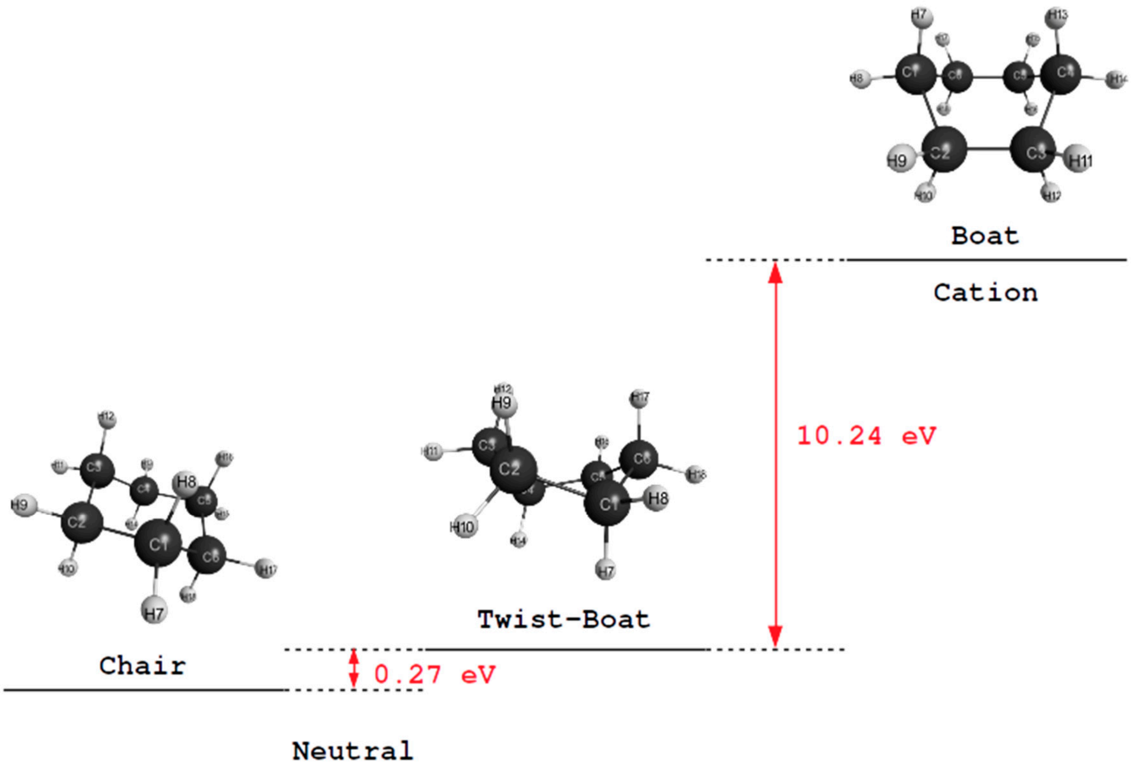

Table S2. The calculated vertical excitation energies and oscillator strengths (TD-DFT/CAMB3LYP/aug-cc-pVTZ) of *chair* cyclohexane (energies in eV). See text for details.

		cyclohexane		
State (C_{2h})	State (D_{3d})	E (eV)	f_L	Dominant excitations
A_g	E_g	7.681	0	HOMO->LUMO (91%)
B_g	E_g	7.681	0	H-1->LUMO (91%)
B_u	E_u	8.025	0.000171	HOMO->L+1 (90%)
A_u	E_u	8.026	0.000172	H-1->L+1 (90%)
A_g	A_{1g}	8.112	0	H-2->LUMO (83%)
B_u	A_{2u}	8.147	0.002014	H-1->L+2 (44%), HOMO->L+3 (44%)
A_u	E_u	8.198	0.034207	HOMO->L+2 (43%), H-1->L+3 (43%)
B_u	E_u	8.198	0.034198	H-1->L+2 (43%), HOMO->L+3 (43%)
A_u	A_{1u}	8.234	0	HOMO->L+2 (43%), H-1->L+3 (44%)
A_u	E_u	8.482	0.003019	H-3->LUMO (30%), H-2->L+2 (54%)
B_u	E_u	8.482	0.003035	H-4->LUMO (30%), H-2->L+3 (54%)
B_u	A_{2u}	8.487	0.221001	H-2->L+1 (90%)
B_g	A_{2g}	8.804	0	HOMO->L+4 (44%), H-1->L+5 (42%)
A_g	E_g	8.808	0	H-1->L+4 (41%), HOMO->L+5 (41%)
B_g	E_g	8.808	0	HOMO->L+4 (40%), H-1->L+5 (42%)
A_g	A_{1g}	8.870	0	H-1->L+4 (44%), HOMO->L+5 (44%)
A_g	E_g	8.946	0	HOMO->L+6 (82%)
B_g	E_g	8.946	0	H-1->L+6 (82%)
A_g	A_{1g}	8.964	0	H-1->L+7 (46%), HOMO->L+8 (46%)
B_g	E_g	8.990	0	HOMO->L+7 (44%), H-1->L+8 (44%)

A_g	E_g	9.990	0	H-1->L+7 (44%), HOMO->L+8 (44%)
B_g	A_{2g}	9.010	0	HOMO->L+7 (44%), H-1->L+8 (44%)
B_g	E_g	9.020	0	H-3->L+1 (50%), H-2->L+7 (16%)
A_g	E_g	9.020	0	H-4->L+1 (50%), H-2->L+8 (16%)
A_g	E_g	9.135	0	H-4->L+1 (16%), H-3->L+2 (13%), H-4->L+3 (13%), H-2->L+5 (39%)
B_g	E_g	9.136	0	H-3->L+1 (16%), H-4->L+2 (13%), H-3->L+3 (13%), H-2->L+4 (39%)
A_u	E_u	9.137	0.003653	H-3->LUMO (59%), H-2->L+2 (32%)
B_u	E_u	9.137	0.003655	H-4->LUMO (59%), H-2->L+3 (32%)
A_g	A_{1g}	9.331	0	H-3->L+2 (14%), H-4->L+3 (14%), H-2->L+6 (61%)
B_g	A_{2g}	9.501	0	H-4->L+2 (44%), H-3->L+3 (44%)
B_g	E_g	9.589	0	H-3->L+1 (22%), H-2->L+7 (70%)
A_g	E_g	9.589	0	H-4->L+1 (22%), H-2->L+8 (70%)
A_g	A_{1g}	9.635	0	H-3->L+2 (27%), H-4->L+3 (27%), H-2->L+6 (28%)
B_u	E_u	9.677	0.094041	HOMO->L+9 (83%)
A_u	E_u	9.677	0.094079	H-1->L+9 (83%)
B_g	E_g	9.705	0	H-4->L+2 (24%), H-3->L+3 (25%), H-2->L+4 (41%)
A_g	E_g	9.706	0	H-3->L+2 (24%), H-4->L+3 (24%), H-2->L+5 (41%)
B_u	A_{2u}	9.748	0.000047	H-1->L+10 (34%), HOMO->L+11 (34%)
A_u	A_{1u}	9.760	0	H-5->LUMO (15%), HOMO->L+10 (27%), H-1->L+11 (24%)
A_u	E_u	9.766	0.000238	HOMO->L+10 (30%), H-1->L+11 (33%)
B_u	E_u	9.766	0.000235	H-1->L+10 (31%), HOMO->L+11 (32%)
A_u	A_{1u}	9.893	0	H-5->LUMO (51%), HOMO->L+10 (10%), H-1->L+11 (10%)
B_u	A_{2u}	9.905	0.047456	H-3->L+4 (28%), H-4->L+5 (28%), H-2->L+9 (29%)
A_u	A_{1u}	9.959	0	H-5->LUMO (12%), H-4->L+4 (29%), H-3->L+5 (29%), H-2->L+16 (10%)
B_u	E_u	9.978	0.003086	H-4->L+6 (38%), H-2->L+11 (18%)
A_u	E_u	9.978	0.002958	H-3->L+6 (38%), H-2->L+10 (18%)

A_u	E_u	10.014	0.106166	H-6->LUMO (28%), H-1->L+14 (33%), H-1->L+17 (13%)
B_u	E_u	10.014	0.104961	H-7->LUMO (28%), HOMO->L+14 (35%), HOMO->L+17 (13%)
B_u	E_u	10.039	0.030083	H-7->LUMO (17%), H-4->L+6 (10%), HOMO->L+14 (11%), HOMO->L+17 (11%)
A_u	E_u	10.040	0.028772	H-6->LUMO (17%), H-1->L+14 (15%), H-1->L+17 (12%)
B_u	A_{2u}	10.060	0.009169	H-1->L+12 (18%), HOMO->L+13 (18%)
B_u	A_{2u}	10.091	0.022573	H-3->L+7 (18%), H-4->L+8 (29%), H-2->L+14 (11%)
A_u	E_u	10.094	0.016806	H-4->L+7 (31%), H-3->L+8 (31%)
B_u	E_u	10.094	0.017719	H-3->L+7 (36%), H-4->L+8 (26%)
A_u	A_{1u}	10.123	0.000391	HOMO->L+12 (27%), H-1->L+13 (21%), H-1->L+18 (10%), HOMO->L+19 (13%)
A_u	E_u	10.129	0.029875	H-1->L+13 (13%), HOMO->L+16 (45%)
B_u	E_u	10.129	0.029374	H-1->L+16 (45%)
B_g	E_g	10.157	0	H-1->L+15 (78%)
A_g	E_g	10.157	0	HOMO->L+15 (78%)
A_u	Eu	10.165	0.000395	H-4->L+4 (39%), H-3->L+5 (39%)
B_u	E_u	10.165	0.000358	H-3->L+4 (39%), H-4->L+5 (39%)
B_u	E_u	10.170	0.00553	H-7->LUMO (15%), H-1->L+16 (36%)
A_u	E_u	10.170	0.005672	H-6->LUMO (15%), HOMO->L+16 (37%)
B_g	A_{2g}	10.184	0	H-5->L+1 (82%)
A_u	E_u	10.217	0.011131	H-1->L+14 (26%), H-1->L+17 (51%)
B_u	E_u	10.217	0.011318	HOMO->L+14 (26%), HOMO->L+17 (50%)
A_u	A_{1u}	10.273	0	H-4->L+7 (45%), H-3->L+8 (45%)
B_g	E_g	10.348	0	H-6->L+1 (83%)
A_g	E_g	10.348	0	H-7->L+1 (83%)
Bu	A_{2u}	10.389	0.002959	HOMO->L+18 (32%), H-1->L+19 (34%)
A_u	E_u	10.402	0.033565	H-3->L+6 (25%), H-2->L+10 (46%), H-2->L+12 (10%)
B_u	E_u	10.402	0.033467	H-4->L+6 (25%), H-2->L+11 (46%), H-2->L+13 (10%)

B_g	E_g	10.410	0	H-5->L+3 (61%)
A_g	E_g	10.410	0	H-5->L+2 (60%)
B_u	A_{2u}	10.443	0.006024	H-3->L+4 (14%), H-4->L+5 (14%), H-2->L+9 (47%)
A_g	E_g	10.461	0	H-6->L+2 (23%), H-7->L+3 (24%), H-1->L+20 (14%), HOMO->L+21 (13%)
B_g	E_g	10.462	0	HOMO->L+20 (34%), H-1->L+21 (31%)
A_u	E_u	10.481	0.002121	HOMO->L+12 (13%), H-1->L+18 (21%), HOMO->L+19 (36%)
B_u	E_u	10.481	0.002179	H-1->L+12 (10%), HOMO->L+13 (11%), HOMO->L+18 (29%), H-1->L+19 (28%)
A_u	A_{1u}	10.487	0.000039	H-1->L+13 (15%), H-1->L+18 (37%), HOMO->L+19 (22%)
A_g	E_g	10.549	0	H-6->L+2 (13%), H-7->L+3 (11%), H-1->L+20 (27%), HOMO->L+21 (26%)
B_g	E_g	10.550	0	H-7->L+2 (12%), H-6->L+3 (13%), HOMO->L+20 (25%), H-1->L+21 (27%)
A_g	E_g	10.593	0	H-6->L+2 (26%), H-5->L+2 (13%), H-7->L+3 (27%), HOMO->L+22 (12%)
B_g	E_g	10.594	0	H-7->L+2 (26%), H-6->L+3 (25%), H-5->L+3 (13%), H-1->L+22 (12%)
A_g	A_{1g}	10.610	0	H-2->L+15 (70%)
A_g	A_{1g}	10.639	0	H-6->L+2 (15%), H-7->L+3 (15%), H-1->L+20 (27%), HOMO->L+21 (30%)
A_u	E_u	10.646	0.00459	H-2->L+10 (19%), H-2->L+12 (53%)
B_u	E_u	10.649	0.004624	H-2->L+11 (18%), H-2->L+13 (53%)
B_u	A_{2u}	10.654	0.004345	H-2->L+9 (10%), H-2->L+14 (67%)
B_g	A_{2g}	10.729	0	H-7->L+2 (39%), H-6->L+3 (38%)
B_u	A_{2u}	10.751	0.173676	H-2->L+17 (87%)
A_g	E_g	10.756	0	HOMO->L+22 (68%)
B_g	E_g	10.756	0	H-1->L+22 (67%)
A_u	A_{1u}	10.854	0	H-2->L+16 (75%)
B_g	E_g	10.877	0	H-3->L+9 (30%), H-2->L+20 (27%)
A_g	E_g	10.878	0	H-4->L+9 (31%), H-2->L+21 (26%)
A_g	E_g	10.907	0	H-4->L+9 (30%), H-3->L+10 (13%), H-4->L+11 (13%)
B_g	E_g	10.907	0	H-3->L+9 (30%), H-4->L+10 (13%), H-3->L+11 (13%), H-3->L+14 (10%)

A_g	A_{1g}	10.937	0	H-1->L+23 (42%), HOMO->L+24 (41%)
A_u	A_{1u}	10.960	0.017794	H-2->L+19 (70%)

Table S3. Harmonic frequencies computed at the DFT/CAMB3LYP/ aug-cc-pVTZ level of theory for *chair* cyclohexane neutral electronic ground-state, compared with experimental data.

$\tilde{X}\ ^1A_{1g}$								
this work		mode	C_{2h}	mode	D_{3d}	*	Exp. [2] cm^{-1}	Assignment
cm^{-1}	eV					eV		
3079.11	0.3817	1	A_g	1	A_{1g}	0.3642	2930	CH_2 s-stretching
3024.79	0.3750	2	A_g	2	A_{1g}	0.3578	2852	CH_2 s-stretching
1521.12	0.1886	3	A_g	3	A_{1g}	0.1799	1465	CH_2 scissoring
1202.76	0.1491	4	A_g	4	A_{1g}	0.1423	1157	CH_2 rocking
820.87	0.1018	5	A_g	5	A_{1g}	0.0971	802	CC stretching
392.58	0.0487	6	A_g	6	A_{1g}	0.0464	383	CCC bending + CC torsion
1386.56	0.1719	7	A_u	7	A_{1u}	0.1640	1383	CH_2 twisting
1143.16	0.1417	8	A_u	8	A_{1u}	0.1352	1157	CH_2 wagging
1118.90	0.1387	9	A_u	9	A_{1u}	0.1323	1057	CC stretching + CC torsion
1364.93	0.1692	10	B_g	10	A_{2g}	0.1614	1437	CH_2 wagging
1081.29	0.1341	11	B_g	11	A_{2g}	0.1279	1090	CH_2 twisting
3084.33	0.3824	12	B_u	12	A_{2u}	0.3648	2915	CH_2 a-stretching
3032.94	0.3760	13	B_u	13	A_{2u}	0.3587	2860	CH_2 s-stretching
1506.44	0.1868	14	B_u	14	A_{2u}	0.1782	1437	CH_2 scissoring
1061.46	0.1316	15	B_u	15	A_{2u}	0.1255	1030	CH_2 rocking
539.13	0.0668	16	B_u	16	A_{2u}	0.0638	523	CCC stretching
3077.12	0.3815	17	A_g	17	E_g	0.3640	2930	CH_2 a-stretching
3077.12	0.3815	18	B_g					
3029.87	0.3756	19	A_g	18	E_g	0.3584	2897	CH_2 s-stretching
3029.87	0.3756	20	B_g					
1495.03	0.1854	21	A_g	19	E_g	0.1768	1443	CH_2 scissoring
1495.03	0.1854	22	B_g					
1395.44	0.1730	23	A_g	20	E_g	0.1650	1347	CH_2 wagging
1395.44	0.1730	24	B_g					
1305.36	0.1618	25	A_g	21	E_g	0.1544	1266	CH_2 twisting
		26	B_g					
1051.82	0.1304	27	A_g	22	E_g	0.1244	1027	CC stretching
		28	B_g					
811.39	0.1006	29	A_g	23	E_g	0.0960	785	CH_2 rocking
		30	B_g					
435.75	0.0540	31	A_g	24	E_g	0.0515	426	CCC bending + CC torsion
		32	B_g					
3075.54	0.3813	33	A_u	25	E_u	0.3638	2933	CH_2 a-stretching
		34	B_u					
3023.70	0.3749	35	A_u	26	E_u	0.3576	2863	CH_2 s-stretching
		36	B_u					
1500.91	0.1861	37	A_u	27	E_u	0.1775	1457	CH_2 scissoring
		38	B_u					
1399.99	0.1736	39	A_u	28	E_u	0.1656	1355	CH_2 wagging
		40	B_u					
1297.67	0.1609	41	A_u	29	E_u	0.1535	1261	CH_2 twisting
		42	B_u					
932.13	0.1156	43	A_u	30	E_u	0.1102	907	CH_2 rocking

		44	B_u					
882.48	0.1094	45	A_u	31	E_u	0.1044	863	CC stretching
		46	B_u					
242.99	0.0301	47	A_u	32	E_u	0.0287	284	CCC bending + CC torsion
		48	B_u					

* energy with scaling factor correction (0.954) for CAMB3LYP [3]

Table S4. Harmonic frequencies computed at the DFT/CAMB3LYP/aug-cc-pVTZ level of theory for cationic *boat-axial* ground-state cyclohexane.

$\tilde{X}\ ^2A$				
this work		*	mode	assignment
cm^{-1}	eV	eV		
3185.81	0.3950	0.3768	ν_1(a)	CH_2 s-stretching
3178.29	0.3940	0.3759	ν_2(a)	CH_2 a-stretching
3149.84	0.3905	0.3726	ν_3(a)	CH_2 s-stretching
3147.81	0.3903	0.3723	ν_4(a)	CH s-stretching
3137.03	0.3889	0.3711	ν_5(a)	CH + CH_2 s-stretching
3135.60	0.3888	0.3709	ν_6(a)	CH a-stretching
3130.25	0.3881	0.3702	ν_7(a)	CH_2 s-stretching
3117.34	0.3865	0.3687	ν_8(a)	CH_2 s-stretching
2949.26	0.3656	0.3488	ν_9(a)	CH_2 s-stretching
2909.64	0.3607	0.3442	ν_{10}(a)	CH a-stretching
2906.20	0.3603	0.3437	ν_{11}(a)	CH a-stretching
2883.37	0.3575	0.3410	ν_{12}(a)	CH a-stretching
1525.92	0.1892	0.1805	ν_{13}(a)	CH_2 scissoring
1492.66	0.1851	0.1766	ν_{14}(a)	CH_2 scissoring
1463.08	0.1814	0.1731	ν_{15}(a)	CH_2 scissoring
1462.98	0.1814	0.1730	ν_{16}(a)	CH_2 scissoring
1444.36	0.1791	0.1708	ν_{17}(a)	CH_2 scissoring
1439.32	0.1784	0.1702	ν_{18}(a)	CH_2 scissoring
1338.24	0.1659	0.1583	ν_{19}(a)	CH_2 wagging
1329.57	0.1648	0.1573	ν_{20}(a)	CH_2 wagging
1308.70	0.1623	0.1548	ν_{21}(a)	CH_2 wagging
1279.97	0.1587	0.1514	ν_{22}(a)	CH_2 wagging
1277.96	0.1584	0.1512	ν_{23}(a)	CH_2 wagging
1246.29	0.1545	0.1474	ν_{24}(a)	CH_2 twisting
1202.96	0.1491	0.1423	ν_{25}(a)	CH_2 twisting
1190.23	0.1476	0.1408	ν_{26}(a)	CH_2 wagging
1151.36	0.1427	0.1362	ν_{27}(a)	CH_2 rocking
1111.69	0.1378	0.1315	ν_{28}(a)	CC stretching + CC torsion
1105.92	0.1371	0.1308	ν_{29}(a)	CH_2 wagging
1058.61	0.1312	0.1252	ν_{30}(a)	CH_2 twisting
1009.94	0.1252	0.1195	ν_{31}(a)	CH_2 twisting
979.83	0.1215	0.1159	ν_{32}(a)	CCC deformation
947.90	0.1175	0.1121	ν_{33}(a)	CC stretching
943.17	0.1169	0.1116	ν_{34}(a)	CC stretching
903.36	0.1120	0.1069	ν_{35}(a)	CH_2 rocking
831.30	0.1031	0.0983	ν_{36}(a)	CC stretching
819.23	0.1016	0.0969	ν_{37}(a)	CC stretching
744.97	0.0924	0.0881	ν_{38}(a)	CCC deformation + CC stretching
673.68	0.0835	0.0797	ν_{39}(a)	CH_2 rocking
615.80	0.0763	0.0728	ν_{40}(a)	CC stretching
578.37	0.0717	0.0684	ν_{41}(a)	CH_2 rocking
463.23	0.0574	0.0548	ν_{42}(a)	CCC bending + CC torsion
429.70	0.0533	0.0508	ν_{43}(a)	CCC bending + CC torsion
373.80	0.0463	0.0442	ν_{44}(a)	CCC stretching + CC torsion
310.83	0.0385	0.0368	ν_{45}(a)	CCC bending + CC torsion

300.16	0.0372	0.0355	ν_{46}(a)	CCC bending + CC torsion
170.68	0.0212	0.0202	ν_{47}(a)	CCC bending + CC torsion
76.61	0.0095	0.0091	ν_{48}(a)	CCC bending + CC torsion

* energy with scaling factor correction (0.954) for CAMB3LYP [3].

References

(1) Bode, B. M.; Gordon, M. S. MacMolPlt: A Graphical User Interface for GAMESS. *J. Mol. Graphics Mod.* **1998**, *16*, 133–138.

(2) Shimanouchi, T. Tables of Molecular Vibrational Frequencies. National Bureau of Standards 1967.

(3) Kashinski, D. O.; Chase, G. M.; Nelson, R. G.; Di Nallo, O. E.; Scales, A. N.; Vanderley, D. L.; Byrd, E. F. C. Harmonic Vibrational Frequencies: Approximate Global Scaling Factors for TPSS, M06, and M11 Functional Families Using Several Common Basis Sets. *J. Phys. Chem. A* **2017**, *121*, 2265–2273. https://doi.org/10.1021/acs.jpca.6b12147.
